# Supplementary material for: Robust multicentre detection and classification of colorectal liver metastases on CT: application of foundation models
Source: NPJ Precis Oncol. 2026 May 27;10:326. doi: 10.1038/s41698-026-01482-2 (PMC13493793; doi:10.1038/s41698-026-01482-2)
Supplement: Supplementary file 1 [file 41698_2026_1482_MOESM1_ESM.docx]

# Supplementary material

#### **Effect of ComBat Harmonization on UMedPT Deep Features**

To evaluate whether scanner- or site-specific effects influenced the deep features extracted from the UMedPT foundation model, feature-level harmonization was performed using the ComBat method [[53,54]](https://paperpile.com/c/VyV8dT/Zlqa3+sUXJZ). Harmonization was applied across centres (KAU, GUMED, UMEA, UNIPI) using the open-source neuroCombat implementation, with parameters estimated from the training set and transferred to validation and test data to prevent data leakage.

Following harmonization, the logistic regression model was retrained using the ComBat-adjusted features. On the EuCanImage test set, the model achieved a ROC AUC of 0.72 (95% CI: 0.67–0.76) and a balanced accuracy of 0.67 (95% CI: 0.63–0.70), which was lower than the baseline LR model trained on unharmonized UMedPT deep features (AUC = 0.81, Table 3). These results indicate that harmonization did not improve performance, suggesting that the pretrained foundation model had already learned domain-invariant representations robust to inter-site variability.

Consequently, the unharmonized UMedPT features were retained for all downstream analyses. Nonetheless, performing this comparison demonstrates adherence to reproducibility and harmonization principles recommended in the Radiomics Quality Score (RQS) 2.0 framework [[43]](https://paperpile.com/c/VyV8dT/ROcdS).

***Supplementary Table 1.*** *Baseline clinical characteristics of the EuCanImage CRLM cohort, stratified by the contributing centre. Values are reported as n (%) unless otherwise stated. These variables, although not directly analyzed in the main text, provide context on comorbidities and liver pathologies within the cohort.*

|  | **KAUNOS** | **GUMED** | **UMEA** | **UNIPI** |
| --- | --- | --- | --- | --- |
| ***Clinical variables*** |  |  |  |  |
| **Diagnosis** |  |  |  |  |
| Histopathology | 59 (12.0%) | 75 (8.8%) | - | - |
| Imaging | 431 (88.0%) | 773 (91.2%) | - | 698 (100.0%) |
| **Comorbidity** |  |  |  |  |
| Yes | 141 (28.8%) | 196 (23.1%) | - | 7 (1.0%) |
| No | 338 (69.0%) | 286 (33.7%) | - | 6 (0.9%) |
| unknown | 11 (2.2%) | 366 (43.2%) | - | 685 (98.1%) |
| **Biliary cystadenoma** |  |  |  |  |
| Yes | - | - | - | 1 (0.1%) |
| No | 191 (39.0%) | 286 (33.7%) | - | 41 (5.9%) |
| unknown | 299 (61.0%) | 562 (66.3%) | - | 638 (91.4%) |
| **Biloma** |  |  |  |  |
| Yes | - | 2 (0.2%) | - | - |
| No | 193 (39.4%) | 286 (33.7%) | - | 42 (6.0%) |
| unknown | 297 (60.6%) | 560 (66.0%) | - | 656 (94.0%) |
| **Cholangiocarcinomas**  **(bile duct cancers)** |  |  |  |  |
| Yes | 3 (0.6%) | 2 (0.2%) | - | - |
| No | 190 (38.8%) | 286 (33.7%) | - | 42 (6.0%) |
| unknown | 297 (60.6%) | 560 (66.0%) | - | 656 (94.0%) |
| **Dysplastic liver nodule** |  |  |  |  |
| Yes | 7 (1.4%) | - | - | - |
| No | 185 (37.8%) | 286 (33.7%) | - | 42 (6.0%) |
| unknown | 298 (60.8%) | 562 (66.3%) | - | 656 (94.0%) |
| **Fibrolamellar hepatocellular carcinoma** |  |  |  |  |
| Yes | - | - | - | - |
| No | 192 (39.2%) | 286 (33.7%) | - | 42 (6.0%) |
| unknown | 298 (60.8%) | 562 (66.3%) | - | 656 (94.0%) |
| **Focal hepatic steatosis** |  |  |  |  |
| Yes | 10 (2.0%) | 31 (3.7%) | - | - |
| No | 180 (36.7%) | 286 (33.7%) | - | 42 (6.0%) |
| unknown | 300 (61.2%) | 531 (62.6%) | - | 656 (94.0%) |
| **Focal nodular hyperplasia** |  |  |  |  |
| Yes | 1 (0.2%) | 2 (0.2%) | - | - |
| No | 192 (39.2%) | 286 (33.7%) | - | 42 (6.0%) |
| unknown | 297 (60.6%) | 560 (66.0%) | - | 656 (94.0%) |
| **Hemangioma** |  |  |  |  |
| Yes | 44 (9.0%) | 38 (4.5%) | - | 3 (0.4%) |
| No | 149 (30.4%) | 286 (33.7%) | - | 41 (5.9%) |
| unknown | 297 (60.6%) | 524 (61.8%) | - | 656 (94.0%) |
| **Hepatic abscess** |  |  |  |  |
| Yes | 2 (0.4%) | 2 (0.2%) | - | - |
| No | 191 (39.0%) | 286 (33.7%) | - | 42 (6.0%) |
| unknown | 297 (60.6%) | 560 (66.0%) | - | 656 (94.0%) |
| **Hepatic adenoma** |  |  |  |  |
| Yes | 2 (0.4%) | - | - | - |
| No | 191 (39.0%) | 286 (33.7%) | - | 42 (6.0%) |
| unknown | 297 (60.6%) | 562 (66.3%) | - | 656 (94.0%) |
| **Hepatic angiosarcoma** |  |  |  |  |
| Yes | - | - | - | - |
| No | 193 (39.4%) | 286 (33.7%) | - | 42 (6.0%) |
| unknown | 297 (60.6%) | 562 (66.3%) | - | 656 (94.0%) |
| **Hepatic hydatid cyst** |  |  |  |  |
| Yes | 1 (0.2%) | - | - | - |
| No | 192 (39.2%) | 286 (33.7%) | - | 42 (6.0%) |
| unknown | 297 (60.6%) | 562 (66.3%) | - | 656 (94.0%) |
| **Hepatocellular carcinoma** |  |  |  |  |
| Yes | 3 (0.6%) | 1 (0.1%) | - | - |
| No | 190 (38.8%) | 286 (33.7%) | - | 20 (2.9%) |
| unknown | 297 (60.6%) | 561 (66.2%) | - | 678 (97.2%) |
| **Liver cirrhosis** |  |  |  |  |
| Yes | 27 (5.5%) | 3 (0.4%) | - | 1 (0.1%) |
| No | 166 (33.9%) | 286 (33.7%) | - | 41 (5.9%) |
| unknown | 297 (60.6%) | 559 (65.9%) | - | 656 (94.0%) |
| **Primary hepatic lymphoma** |  |  |  |  |
| Yes | - | - | - | - |
| No | 193 (39.4%) | 286 (33.7%) | - | 42 (6.0%) |
| unknown | 297 (60.6%) | 562 (66.3%) | - | 656 (94.0%) |
| **Simple hepatic cysts** |  |  |  |  |
| Yes | 86 (17.6%) | 183 (21.6%) | - | 5 (0.7%) |
| No | 104 (21.2%) | 286 (33.7%) | - | 38 (5.4%) |
| unknown | 300 (61.2%) | 379 (44.7%) | - | 655 (93.9%) |
| **Transient hepatic**  **attenuation differences** |  |  |  |  |
| Yes | - | - | - | - |
| No | 191 (39.0%) | 286 (33.7%) | - | 42 (6.0%) |
| unknown | 299 (61.0%) | 562 (66.3%) | - | 656 (94.0%) |
| **Pathological T staging (primary tumor)** |  |  |  |  |
| 0 (pTX) | 1 (0.2%) | - | - |  |
| 1 (pT0) | - | 1 (0.1%) | - |  |
| 3 (pT1) | 1 (0.2%) | 3 (0.4%) | - | 6 (0.9%) |
| 4 (pT2) | 8 (1.6%) | 16 (1.9%) | - | 7 (1.0%) |
| 5 (pT3) | 59 (12.0%) | 107 (12.6%) | - | 19 (2.7%) |
| 6 (pT4) | 9 (1.8%) | 6 (0.7%) | - | 4 (0.6%) |
| 7 (pT4a) | 17 (3.5%) | 29 (3.4%) | - | 5 (0.7%) |
| 8 (pT4b) | 3 (0.6%) | 9 (1.1%) | - | 3 (0.4%) |
| unknown | 392 (80.0%) | 677 (79.8%) | - | 654 (93.7%) |
| **Clinical T staging (primary tumor)** |  |  |  |  |
| 0 (cTX) | 31 (6.3%) | 90 (10.6%) | 3 (0.7%) | 1 (0.1%) |
| 3 (cT1) | 1 (0.2%) | 2 (0.2%) | - | - |
| 4 (cT2) | 11 (2.2%) | 10 (1.2%) | 84 (20.9%) | - |
| 5 (cT3) | 60 (12.2%) | 54 (6.4%) | 208 (51.9%) | 19 (2.7%) |
| 6 (cT4) | 22 (4.5%) | 20 (2.4%) | 93 (23.2%) | 8 (1.1%) |
| 7 (cT4a) | 12 (2.4%) | - | - | 2 (0.3%) |
| 8 (cT4b) | 4 (0.8%) | 6 (0.7%) | - | 4 (0.6%) |
| unknown | 349 (71.2%) | 666 (78.5%) | 13 (3.2%) | 664 (95.1%) |
| **Pathological N staging (primary tumor)** |  |  |  |  |
| 0 (pNX) | 2 (0.4%) | 1 (0.1%) | - | - |
| 1 (pN0) | 23 (4.7%) | 46 (5.4%) | - | 7 (1.0%) |
| 2 (pN1) | 48 (9.8%) | 66 (7.8%) | - | 17 (2.4%) |
| 3 (pN2) | 25 (5.1%) | 58 (6.8%) | - | 12 (1.7%) |
| unknown | 392 (80.0%) | 677 (79.8%) | - | 662 (94.8%) |
| **Clinical N staging (primary tumor)** |  |  |  |  |
| 0 (cNX) | 35 (7.1%) | 97 (11.4%) | - | - |
| 1 (cN0) | 12 (2.4%) | 14 (1.7%) | - | 10 (1.4%) |
| 2 (cN1) | 28 (5.7%) | 36 (4.2%) | - | 14 (2.0%) |
| 3 (cN2) | 66 (13.5%) | 35 (4.1%) | - | 18 (2.6%) |
| unknown | 349 (71.2%) | 666 (78.5%) | - | 656 (94.0%) |
| **Pathological M staging (primary tumor)** |  |  |  |  |
| 0 (pM0) | 1 (0.2%) | 137 (16.2%) | - | 6 (0.9%) |
| 1 (pM1) | 63 (12.9%) | 30 (3.5%) | - | 21 (3.0%) |
| 2 (pM1a) | 5 (1.0%) | 1 (0.1%) | - | 14 (2.0%) |
| 3 (pM1b) | - | 3 (0.4%) | - | 1 (0.1%) |
| 4 (pM1c) | - | - | - | 1 (0.1%) |
| unknown | 421 (85.9%) | 677 (79.8%) | - | 655 (93.8%) |
| **Clinical M staging (primary tumor)** |  |  |  |  |
| 0 (cM0) | 4 (0.8%) | 112 (13.2%) | - | 5 (0.7%) |
| 1 (cM1) | 155 (31.6%) | 46 (5.4%) | - | 16 (2.3%) |
| 2 (cM1a) | 3 (0.6%) | 4 (0.5%) | - | 2 (0.3%) |
| 3 (cM1b) | 5 (1.0%) | 7 (0.8%) | - | 5 (0.7%) |
| 4 (cM1c) | 1 (0.2%) | 13 (1.5%) | - | 3 (0.4%) |
| unknown | 322 (65.7%) | 666 (78.5%) | - | 667 (95.6%) |

***Supplementary Figure 1.*** *Calibration curve of the best-performing model on the EuCanImage test set. The diagonal gray dashed line indicates perfect calibration. The solid line shows observed versus predicted probabilities averaged across the prospective validation folds. The model achieved a Brier score of 0.171, calibration slope = 3.40, and intercept = 0.15.*

***Supplementary Table 2.*** *Detailed Radiomics Quality Score (RQS 2.0) evaluation for the proposed multi-center CRLM classification–detection framework. The study achieved an RQS 2.0 score of 28/35 (Radiomics Readiness Level 6), reflecting strong methodological transparency, validation, and interpretability consistent with the RQS 2.0 framework*

| No. | Criteria | Selected Option | Points | Explanation |
| --- | --- | --- | --- | --- |
| **RRL 1 - Foundational Exploration** | | | | |
| 1 | Unmet Clinical Need – Unmet clinical need (UCN) defined.  ● UCN is agreed upon and defined by more than one center.  ● UCN is defined using an established consensus method such as the Delphi method. | Implemented: Delphi method (+2) | 2 | UCN defined and endorsed via consensus across 4 EuCanImage centres, reflecting multi-centre agreement on UCN in CRLM classification and detection |
| 2 | Hardware Description – Detailed description of the imaging hardware used, including model, manufacturer, and technical specifications. | Not implemented | 0 | Detailed scanner/hardware metadata unavailable for the participating centres |
| 3 | Image Protocol Quality – Five levels of image protocol quality for TRIAC:  ● Level 0: Protocol not formally approved.  ● Level 1: Approved with a reference number in the institutional archive.  ● Level 2: Approved with formal quality assurance (recommended minimum for prospective trials).  ● Level 3: Established internationally; published in guidelines and peer-reviewed papers.  ● Level 4: Future proof (follows TRIAC Level 3, FAIR principles, retains raw data). | Not implemented | 0 | No formal or standardized imaging protocol across centres; institutional approval documentation not available |
| 4 | Inclusion and Exclusion Criteria – Detailed criteria for patient selection in studies, including rationale. | Implemented (+1) | 1 | Detailed inclusion/exclusion criteria were defined and are documented in the Methods (Dataset) section |
| 5 | Diversity and Distribution – Identify potential biases before the project (demographics, socioeconomic, geographic, medical profiles). | Implemented (+1) | 1 | Patient demographic and acquisition heterogeneity were assessed (see Table 2) |
| **RRL 2 - Data Preparation** | | | | |
| 7 | Preprocessing of Images – Apply steps to standardize images with clear reasoning. | Implemented (+1) | 1 | Preprocessing steps (resampling, normalization, cropping) are fully described in the Methods section |
| 8 | Harmonization – Use image-level (e.g. CycleGANs) or feature-level (e.g. ComBat) harmonization techniques. | Implemented (+1) | 1 | Feature-level harmonization via ComBat applied across sites to mitigate scanner/site bias |
| 10 | Automatic Segmentation – Use an automated segmentation algorithm for ROI definition. | Implemented (+1) | 1 | Automated segmentation was performed using TotalSegmentator for liver ROI localization (Methods) |
| **RRL 3 - Prototype Model Development** | | | | |
| 13 | HCR + DL Combination – Compare and explore the synergistic combination of handcrafted radiomics and deep learning models. | Implemented (+1) | 1 | Combination of handcrafted radiomics (HCR) and deep-learning (DL) features was not feasible due to partial annotations |
| 14 | Multivariable Analysis – Incorporate non‑radiomics features (clinical, genomic, proteomic) to yield a holistic model. | Implemented (+2) | 2 | Non-radiomics (clinical) features (age, gender) were integrated with UMedPT deep features in a logistic regression model |
| **RRL 4 - Internal Validation** | | | | |
| 15 | Single Center Validation – Validation performed on data from the same institute without retraining or adapting the cut-off value. | Implemented (+1) | 1 | Internal site-wise validation was conducted to confirm within-center generalization |
| 16 | Cut-off Analyses – Identify optimal thresholds (e.g., using Youden’s Index) for classification or survival analysis. | Implemented (+1) | 1 | Optimal classification threshold(s) were selected using Youden’s Index and validated on held-out data |
| 17 | Discrimination Statistics – Report discrimination metrics (e.g., ROC curve, sensitivity, specificity) with significance (p-values, CIs).  ● Statistic reported  ● With Resampling method | Resampling method applied (+2) | 2 | Discrimination metrics (ROC, AUC, sensitivity, specificity, 95% CI) were reported, and statistical significance assessed via resampling (e.g. bootstrapping) |
| 18 | Calibration Statistics – Report calibration metrics (e.g., calibration-in-the-large, slope, plots). | Implemented (+1) | 1 | Calibration performance reported via calibration plots and calibration-in-the-large or slope metrics |
| 19 | Failure Mode Analysis – Document model limitations with examples of edge cases. | Implemented (+1) | 1 | Misclassified/edge cases were qualitatively inspected for both internal (EuCanImage) and external (TCIA_CRLM) cohorts to understand failure modes |
| 20 | Open Science and Data – Make code and data publicly available.  ● Open scans (+1)  ● Open segmentations (+1)  ● Open code (+1) | All aspects (+3) | 3 | Scans, segmentations, and code are made publicly available via the EuCanImage platform and GitHub repository |
| **RRL 5 - Capability Testing** | | | | |
| 21 | Multi‑center Validation – Validation with data from multiple institutes ensuring no overlap:  ● One external institute  ● Two or more external institutes  ● Third‑party platform with completely unseen data | One Institute (+1) | 1 | External validation was limited to the TCIA dataset; no other independent sites were used |
| 22 | Comparison with ‘Current Clinical Standard’ – Assess model agreement or superiority versus the current gold standard (e.g., TNM staging). | Implemented (+2) | 2 | Model performance was evaluated against expert radiologic reference labels that serve as the clinical ground truth for CRLM diagnosis, as TNM or pathological staging data were missing across most centres. |
| 23 | Comparison to Previous Work – Compare performance with published HCR signatures or DL algorithms. | Implemented (+1) | 1 | Comparison with existing literature (handcrafted radiomics ) was limited due to incompatible annotation standards and dataset overlap |
| 24 | Potential Clinical Utility – Report on the current and potential clinical application (e.g., decision curve analysis). | Implemented (+2) | 2 | Decision curve analysis (DCA) is reported to illustrate potential clinical usefulness |
| **RRL 6 - Trustworthiness Assessment** | | | | |
| 25 | Explainability – Apply explainability tools (e.g., SHAP for HCR, GradCAM for DL) to clarify model predictions. | Implemented (+1) | 1 | Explainability via Grad-CAM visualizations is reported, highlighting regions contributing to classification decisions |
| 26 | Explainability Evaluation – Conduct qualitative and quantitative evaluations of interpretability methods (e.g., checking consistency to adversarial perturbations). | Not implemented | 0 | Not implemented |
| 27 | Biological Correlates – Detect and discuss biological correlates to deepen understanding of radiomics and underlying biology. | Not implemented | 0 | Biological correlates (e.g. linking imaging features to molecular markers) were not available in the current dataset but is planned for future work |
| 28 | Fairness Evaluation and Mitigation – Evaluate model performance for biases and apply bias correction if needed.  ● Fairness evaluated  ● Bias correction applied | With Bias correction (+2) | 2 | Fairness analyses were performed (e.g. stratification by lesion size or site), and bias mitigation was applied to correct observed imbalances during training of models (SMOTE and Focal loss) |
|  | Total = 28/35 (80%) |  |  |  |

***Supplementary Figure 2.*** *Additional Grad-CAM visualization examples illustrating model attention patterns across different prediction scenarios. Each panel shows the original CT slice (top) and the corresponding Grad-CAM heatmap (bottom). Panels* ***A-B*** *correspond to* ***true positive (TP)*** *predictions where the model focuses on metastatic lesions,* ***C-D*** *show* ***false positive (FP)*** *cases with attention concentrated on non-lesion hepatic or vascular structures,* ***E-F*** *represent* ***true negative (TN)*** *cases with diffuse or background attention, and* ***G-H*** *illustrate* ***false negative (FN)*** *cases where the model fails to sufficiently focus on the lesion region.*

***Supplementary Figure 3.*** *Patient-level classification sensitivity stratified by lesion characteristics in the* ***TCIA_CRLM*** *cohort (****n = 197****).* ***(A)*** *Sensitivity according to the* ***number of lesions per patient*** *(1, 2-3, ≥4).* ***(B)*** *Sensitivity according to the* ***largest lesion diameter per patient*** *(≤2 cm, 2-5 cm, >5 cm). Error bars indicate* ***95% confidence intervals****. Sensitivity increased with both lesion burden and lesion size.*
